# Understanding user communication around loneliness on online forums

**DOI:** 10.1371/journal.pone.0257791

**Published:** 2021-09-23

**Authors:** Anietie Andy

**Affiliations:** Penn Medicine, University of Pennsylvania, Philadelphia, Pennsylvania, United States of America; University of Pisa, ITALY

## Abstract

Increasingly, individuals experiencing loneliness are seeking support on online forums—some of which focus specifically on discussions around loneliness (loneliness forums); loneliness may influence how these individuals communicate in other online forums not focused on loneliness (non-loneliness forums). In order to provide effective and appropriate online interventions around loneliness, it is important to understand how users who publish posts in a loneliness forum communicate in the loneliness forum and non-loneliness forums they belong to. In this paper, using language features, the following analyses are conducted: (1) Posts published on an online loneliness forum on Reddit, */r/Lonely* are compared to posts (published by the same users and around the same time period) on two Reddit online forums i.e. an advice seeking forum, */r/AskReddit* and a forum focused on discussions around depression (depression forum), */r/depression*. (2) Interventions related to loneliness may vary depending on if an individual is lonely and depressed or lonely but not depressed; language use differences in posts published in */r/Lonely* by the following set of users are identified: (a) users who post in both */r/Lonely* and a depression forum and (b) users who post in */r/Lonely* but not in the depression forum. The findings from this work gain new insights, for example: (i) */r/Lonely* users tend to seek advice/ask questions related to relationships in the advice seeking forum, */r/AskReddit* and (ii) users who are members of the loneliness forum but not the depression forum tend to publish posts (on the loneliness forum) on topic themes related to work/job, however, those who are members of the loneliness and depression forums tend to use more words associated with anger, negation, death, and post on topic themes related to affection relative to relationships in their loneliness forum posts. Some of the findings from this work also align with prior work e.g. users who express loneliness in online forums tend to make more reference to self. These findings aid in gaining insights into how users communicate on these forums and their support needs, thereby informing loneliness interventions.

## 1 Introduction

Loneliness is a risk factor for conditions such as depression [1], coronary heart disease, and stroke [2]. Increasingly, individuals experiencing loneliness are seeking support on social media and online forums [3–5], some of which focus on discussions around loneliness (loneliness forum). Loneliness may influence how individuals express themselves in different settings or forums, therefore, in order to provide efficient and appropriate interventions to address loneliness, it is important to understand how users communicate in a loneliness forum compared to other online forums—not focused on loneliness (non-loneliness forums), they belong to.

Reddit, which is an online platform made up of several sub-forums and allows access to user posts in forums they belong to, provides an ideal setting for analyzing and understanding how individuals communicate in various forums they belong to. In this work, a loneliness forum, */r/Lonely* on Reddit is identified and using language features, posts in this forum are analyzed and compared to posts (published by the same users) on an advice seeking forum on Reddit, */r/AskReddit* and a Reddit forum focused on discussions around depression, */r/depression*.

Prior work characterized and described how users respond to individuals who express loneliness on Twitter and an online forum [3–7]; however, prior work did not analyze the differences in language use in posts by the same users in a loneliness forum compared to other forums.

Using social media and online forum data and language features such as the topic modeling algorithm, Latent Dirichlet Allocation (LDA) [8] and the Linguistic Inquiry Word Count (LIWC) [9]—which is a dictionary of psycho-linguistic categories, prior work measured the extent of social supports expressed in online forum posts and determined: (a) how the social supports given and sought changes over time in a COVID-19 online forum [10] and (b) the social supports (expressed in posts) that elicit responses in substance use recovery online forums [11]. Also, using social media and online forum data and LDA and LIWC, prior work predicted patients risk for cardiovascular disease [12], and characterized users expressing loneliness from others who did not express loneliness [3, 7]. Hence, similar to prior work, this work uses the language features, LDA and LIWC to analyze posts published (by the same users) in an online loneliness forum (*/r/Lonely*) and non-loneliness forums (*/r/AskReddit* and */r/depression*) to gain insights into how these users communicate in these forums.

Studies have shown that there is a correlation between depression and loneliness [13, 14], hence interventions around loneliness may differ based on if an individual is lonely and depressed or lonely but not depressed. An analysis is conducted to determine if there is a difference in the way users who are lonely and may be depressed express themselves in a loneliness forum compared to users who are lonely but not depressed. To do this, posts published on the loneliness forum, */r/Lonely* by the following group of users are compared: (i) users who are members of both */r/Lonely* and the depression forum and had published posts in both forums at the same time period and (ii) users who are members of */r/Lonely* but not members of the depression forum.

The findings from this work indicate that these language features reflect some of the support needs and concerns of these users and can provide insights as to how these users communicate on these forums, thereby informing online loneliness interventions.

Specific contributions in this paper are as follows:

- The differences in language use in posts published (by the same users and at the same time period) in a loneliness forum compared to posts in an advice seeking forum and a depression forum, respectively are determined
- Given posts published in a loneliness forum, the differences in language use in the loneliness forum posts by users who are members of both the loneliness forum and a depression forum compared to users who are members of the loneliness forum but not members of the depression forum are determined.

For all the analysis in this paper, the effect sizes are reported using Cohen’s D—which is the standardized difference between two means. Only results with Cohen’s D greater than or equal to a threshold (0.10) are displayed; also, only significant topics after Benjamini-Hochberg p-correction and p < 0.001 are reported. In this work, users are considered active members of a forum if they are members of a forum and have at least one published post in the forum. Reddit posts are made up of two parts: the *title* section—which is a brief description of the post and the *selftext* section—which is a more descriptive section of the post. For all our analysis in this work, the sentences in the *title* and *selftext* sections of posts are combined; hence, when this work refers to a post, it is a combination of the *title* and *selftext* sections.

## 2 Materials and methods

### 2.1 Ethics and privacy

In this work, social media data related to loneliness and depression are analyzed. Due to how sensitive data related to mental health and individual well-being is, precautions were taken to make sure the privacy of the users in the dataset used for this work was respected.

The dataset used for this work is publicly available (as mentioned in section **3**, Google’s BigQuery [15] was used to obtain the dataset). For all the analysis in this work, information from user profiles was not used and no user or any of the moderators in the */r/Lonely*, */r/depression*, and */r/AskReddit* forums were contacted; also no Reddit users or moderators were contacted.

## 3 Related work

Several prior works have shown that some individuals post about their experiences as it relates to healthcare [16, 17] and seek support around their health and well-being on social media platforms such as Twitter and Reddit; data obtained from these platforms have been used to gain insights into the support needs of these users and how they communicate on these forums [11, 12, 17–20].

Prior works [18–20] analyzed posts and comments in an online cancer forum to understand the types of social supports users seek and give on the forum and how these social supports expressed by these users change over the course of their membership in the forum. [18] studied how the messages users in an online cancer forum are exposed to affects their continued involvement in the forum [19]. Studied the roles users of an online cancer forum take on and how these roles change over time [20]. Determined that users in an online cancer forum tended to share more negative information about themselves in their public facing messages compared to private ones. In [12], using data from electronic health records and social media, patients risk for cardiovascular disease was predicted [11]. Analyzed posts published on online substance use recovery forums to determine the types of social support expressed in posts on these forums that elicit responses from members of the forum. In [21–23], social media data was used to gain insights about mental illness and well-being. In [10], the social supports expressed in an online COVID-19 forum were measured and for example, it was determined that over time, users sought more emotional social support in their posts. In [24], data from several online forums focused on mental health discussions were analyzed to characterize the changes in these forums during the beginning of the COVID-19 pandemic; it was found that, for example, posts related to COVID-19 were published on an online Reddit forum focused on discussions around anxiety more than a month before other mental health support groups on Reddit started posting about COVID-19. Also, it was found that in these mental health related forums, there was an increase in posts related to suicide and loneliness during the pandemic.

This work is focused on loneliness; below are related works on loneliness.

Loneliness is becoming a major public health concern [3]; it affects the physical and mental well-being of individuals and is associated with an increased risk of early mortality in older individuals [25, 26]. The feeling of loneliness for a long period of time may lead to depression and suicidal thoughts [27, 28]. As mentioned in section **1**, prior work showed that loneliness is a risk factor for depression [1], coronary heart disease, and stroke [2]. Also, prior work demonstrated that loneliness affects individuals in all age groups [29, 30].

Increasingly, individuals experiencing loneliness are seeking support on social media platforms such as Reddit and Twitter. Several studies have been done to understand and characterize how users express loneliness on social media. In [3], individuals who self-express loneliness on Twitter were characterized and it was determined that these users used more words associated with mental health when compared to a control group. In [5], it was determined that individuals who express more loneliness on social media tend to have less online relationships. In [4], it was determined that tweets in which a user self-declared to be lonely received more responses than the other tweets (by the same user) which did not express loneliness. In [31], it was shown that increased loneliness is associated with more social media use. In [32], it was determined that decreased depression and loneliness was associated with the limited use of social media platforms. [7] studied how users who express the feeling of loneliness in an online loneliness forum communicate in a non-loneliness forum; it was found that in the non-loneliness forum, these users tend to use more words associated with sadness, post more on topics related to relationships, family and friends and mental health when compared to a control group of users.

This work is different from prior work; this work aims to determine the differences in the way users communicate in a loneliness forum compared to other forums in which they belong.

## 4 Dataset

The datasets used for this work were collected from 3 Reddit forums i.e. */r/Lonely*, */r/AskReddit*, and */r/depression*. Below is a description of why these forums were selected.

First, to identify online loneliness forums on Reddit, using Google’s BigQuery (BigQuery) [15]—a data warehouse which includes Reddit datasets, data from forums focused on discussions around loneliness were selected and collected by identifying Reddit forums that included the word *“lonely”* in its name, such as *“/r/iAMlonely”*, *“/r/lonelyheartbeats”*, and *“/r/Lonely”*. It was observed that during the time period in which the data was collected (i.e. between December 2015 and August 2019), */r/Lonely* had more users and significantly more posts compared to the other loneliness forums, which mostly had a few hundred posts with non of the forums having up to 300 posts during the time period in which the data was collected. While combining posts from different online forums might give more data to analyze, prior work determined that different online forums, despite their similarities, differ in terms of the discussions and interests of their members [33]. Hence, in this work, posts published on */r/Lonely* were collected. Specifically, using BigQuery, all posts published between December 2015 and August 2019 in the Reddit forum */r/Lonely* were collected. */r/Lonely* has 217,000 members as of May 2021 and is self-described as *“a sub for all the lonely people. Everyone is welcome here, no matter your age, race, sex, sexuality, relationship status, all that we request is that you be accepting of people, and kind. Any problems at all, please let the moderators know”*.

In order to avoid selecting posts by users who may publish posts in the loneliness forum but not express loneliness, a health professional reviewed the data collected from */r/Lonely* and selected data from users who self-declared to be lonely in a post, for example (rephrased): *“I feel sad and alone; I do not have any real friends”*. Table 1 shows the number of users and posts in the */r/Lonely* dataset.

**Table 1:** Summary of our */r/Lonely dataset*.

Number of users	Number of posts
9,956	15,014

To identify the other Reddit forums in which users in the */r/Lonely* dataset are members of, the user names in the */r/Lonely* dataset were collected; BigQuery was used to search for these user names in all the Reddit forums (i.e. between December 2015 and August 2019) to determine the other forums in which these users are members of and have published posts. While some of these */r/Lonely* users are members of several forums, it was observed that the forums with the highest number of these users as active members are: (i) */r/AskReddit* (an advice seeking forum) with 24% of these users as active members and (ii) */r/depression* (a forum focused on discussions around depression) with 20% of these users as active members. Hence, in this work, posts published by users in */r/Lonely* are compared to posts published by the same users in */r/AskReddit* and */r/depression*, respectively. Below is a description of the datasets used in this work:

### 4.1 Dataset: */r/Lonely* and */r/AskReddit*

From the */r/Lonely* dataset (Table 1), 2,401 users who had published posts in both the */r/Lonely* and */r/AskReddit* forums, respectively were identified; Table 2 shows the summary of posts published by these users.

**Table 2:** Summary of posts published by 2,401 users on */r/Lonely* and */r/AskReddit*.

Forum	Number of posts
*/r/Lonely*	4,001
*/r/AskReddit*	25,833

### 4.2 Dataset: */r/Lonely* and */r/depression*

2,031 users from the */r/Lonely* dataset (Table 1) with published posts on the */r/Lonely* and */r/depression* forums were identified; a summary of these posts is shown in Table 3.

**Table 3:** Summary of posts published by 2,031 users on */r/Lonely* and */r/depression*.

Forum	Number of posts
*/r/Lonely*	4,389
*/r/depression*	9,756

A health professional reviewed the posts from */r/depression* and determined that these posts referenced the users feeling depressed; for example (rephrased): *“I have had anxiety and depression for some time now; I recently sought help but for the past several weeks, its been really bad and it is starting to affect my school work”*.

### 4.3 Dataset: Posts in */r/Lonely* by */r/Lonely* users only compared to */r/Lonely* + */r/depression* users

Table 4 shows the summary of posts published in */r/Lonely* by users who are active members of */r/Lonely* and */r/depression* and users who are active members of */r/Lonely* but not members of */r/depression*.

**Table 4:** Summary of posts published in */r/Lonely* by users who are active members of */r/Lonely* and */r/depression* and users who are active members of */r/Lonely* but not */r/depression*.

	No. of users	No. of posts
*/r/Lonely only*	7,925	10,625
*/r/Lonely + /r/depression*	2,031	4,389

This paper is formatted as follows—using LDA and LIWC: (a) in section 5, the differences in language use in posts published around the same time period in */r/Lonely* and */r/AskReddit*, by the same users is determined, (b) in section 6, the differences in language use in posts (published by the same users) in the */r/Lonely* and */r/depression* datasets are determined, and (c) in section 7, the differences in language use in posts published on */r/Lonely* by users who are active members of both */r/Lonely* and */r/depression* compared to users who are active members of */r/Lonely* but not active members of */r/depression* are determined.

## 5 Compare posts in */r/Lonely* to posts in */r/Askreddit*

Here, LDA and LIWC are used to determine the language use differences in posts published by the same users and around the same time period on */r/Lonely* and */r/AskReddit*, respectively. The dataset from Table 2 was used for the analysis in this section.

### 5.1 LDA

To determine the LDA topics most associated with posts in */r/Lonely* compared to */r/AskReddit* and vice versa, the following steps were carried out: (a) the tokenization tool, HappierFunTokenizer [34]—which is able to identify regular words, emoticons, and variations in word spellings, was used to tokenize/identify the single words in posts in both the */r/Lonely* and */r/AskReddit* datasets (Table 2), (b) after the tokenization of the words, the LDA algorithm implementation by Mallet [35] was used to extract LDA topics. LDA clusters co-occurring words in documents (in this case Reddit posts). LDA, which is a generative model, makes the assumption that topics are made up of a combination of words/tokens and the Reddit posts are made up of a mixture of topics. Given that the words in the Reddit posts are known, Gibbs sampling [36] can be used to estimate the latent variables of the topics. Based on the words associated with a topic, a label can be assigned to the topic; for example, LDA may cluster the words (*January*, *February*, *March*, *April*, *May*) as months of the year. In this section, 20 LDA topics were generated from all the posts described in Table 2 including the */r/Lonely* and */r/AskReddit* posts. To identify the number of topics, similar to prior works [7, 12, 16, 37], the number of LDA topics were varied between 5 and 50 topics; the author reviewed these topics and determined that 20 topics had more coherent themes compared to the others, hence, 20 topics was used for the analysis.

Using the generated 20 LDA topics, the topic themes that are most associated with */r/Lonely* posts when compared with */r/AskReddit* posts and vice versa are identified. Table 5 shows the effect sizes (measured using Cohen’s D) between the most significant topic distributions in posts published in the forum, */r/Lonely* compared to posts in the forum */r/AskReddit*. Similarly, Table 6 shows the effect sizes between the most significant topic distributions in posts published in the forum, */r/AskReddit* when compared to posts in */r/Lonely*.

**Table 5:** Topics associated with */r/Lonely* when compared to */r/AskReddit*.

LDA Topics and highly correlated words	Cohen’s D
feel, love, loneliness, feeling, mind, long, thoughts, found, happy, heart	1.325
i’m, don’t, feel, it’s, i’ve, lonely, freinds, can’t, talk, i’ll	1.308
friends, school, college, high, make, group, year, hang, meet, awkward	0.400
didn’t, told, back, thought, wanted, felt, made, girl, shit, asked	0.359
year, time, day, family, today, back, ago, birthday, lost, relationship	0.192
work, time, job, home, night, day, live, hours, sleep, working,	0.107

**Table 6:** Topics associated with */r/AskReddit* when compared to */r/Lonely*.

LDA Topics and highly correlated words	Cohen’s D
what’s, thing, you’ve, worst, story, heard, happened, weirdest, whats, experience	0.582
song, music, favorite, listen, dog, words, hear, listening, mind, choose	0.491
reddit, post, question, subreddit, internet, comment, questions, phone, comments, media	0.433
movie, favorite, show, tv, game, video, watch, character, book, shows	0.397
world, country, opinion, human, society, America, states, body, war, future	0.394
life, real, made, change, things, dream, moment, world, biggest, important	0.314
food, eat, money, buy, car, place, drink, pay, store, bought	0.299
women, girl, sex, guy, find, dating, date, men, relationship, attractive	0.118

### 5.2 LIWC

In this section, the the differences in the use of LIWC categories between posts published (by the same users) in */r/Lonely* compared to posts published in */r/AskReddit* and vice versa, are determined. LIWC is a dictionary made up of 73 psycho-linguistic categories such as positive and negative emotions, health, and personal pronouns; each of these categories consists of a curated list of words. For all the posts in the dataset (Table 2) used in this section, the proportion of words associated with the LIWC categories in */r/Lonely* posts compared to those in */r/AskReddit* posts are determined. Tables 7 and 8 show the different LIWC categories associated with */r/Lonely* and */r/AskReddit*, respectively.

**Table 7:** Results from LIWC categories associated with posts in */r/Lonely* compared to posts in */r/AskReddit* by the same users.

LIWC category	Cohen’s D
First Person singular pronoun	2.047
Negations	0.578
Sadness	0.549
Personal pronouns	0.430

**Table 8:** Results from LIWC categories associated with posts in */r/AskReddit* compared to posts in */r/Lonely* by the same users.

LIWC category	Cohen’s D
Interrogatives (i.e. Questions)	1.769
Second Person pronouns	1.312
Impersonal pronouns	0.698
Social processes	0.467
Leisure	0.319

### 5.3 Results

It was observed that in the loneliness forum, users tend to express their thoughts and feelings about loneliness, make reference to issues with socializing in school/college, and make reference to topic themes on time relative to relationships (Table 5). Also, in loneliness forum posts, users tend to use more words associated with the LIWC category on sadness and make more reference to self i.e. “first person singular pronouns”(Table 7).

In the advice seeking forum, */r/AskReddit*, users tend to post about songs they listen to and movies they watch, and seek advice/ask questions on topics related to relationships (Table 6). Also in the the advice seeking forum, these users tend to use more words associated with the LIWC categories on socializing and leisure (Table 8).

## 6 Compare posts in */r/Lonely* and */r/depression*

In this section, similar to section **4**, using LDA and LIWC, the aim here is to determine the differences in language use in posts (published by the same users) in the */r/Lonely* and */r/depression* datasets.

### 6.1 LDA

Similar to section **4.1**, using LDA, 20 LDA topics were generated using posts from the */r/Lonely* and */r/depression* datasets (Table 3). From the generated topics, the topics which occur more frequently in posts published in */r/Lonely* compared to posts published in */r/depression* and vice versa were identified. Table 9 shows the effect sizes between the most significant topic distributions in posts published in the forum */r/Lonely* compared to posts published in the */r/depression* forum. Also, Table 10 shows the effect sizes between the most significant topic themes in posts in the */r/depression* forum compared to those in the */r/Lonely* forum.

**Table 9:** Topics associated with */r/Lonely* when compared to */r/depression*.

LDA Topics and highly correlated words	Cohen’s D
love, girl, relationship, girlfriend, guy, sex, women, dating, date, loved	0.367
talk, lonely, post, chat, reddit, i’d, removed, stuff, message, play	0.313
friend, talk, lonely, person, close, hang, talking, real, miss, friendship	0.260

**Table 10:** Topics associated with */r/depression* when compared to */r/Lonely*.

LDA Topics and highly correlated words	Cohen’s D
depression, anxiety, depressed, mental, health, therapy, suicidal, therapist, thoughts, worse	0.409
i’m, don’t, can’t, i’ve, it’s, anymore, i’ll, care, doesn’t, tired	0.313
life, things, happy, end, change, point, hope, past, future, living	0.283
fucking, shit, fuck, hate, die, stupid, fucked, piece, kill, god	0.281
you’re, care, thing, that’s, put, understand, real, read, words, write	0.188
job, work, money, working, live, week, pay, car, jobs, house	0.178
world, human, death, society, understand, hope, suffering, body, pain, matter	0.177
day, sleep, bed, night, home, wake, today, days, eat, morning	0.132

### 6.2 LIWC

Similar to section 5.2, LIWC is used to determine the differences in the use of LIWC categories between posts published in */r/Lonely* compared to posts published in */r/depression*, by the same users. Tables 11 and 12 show the different LIWC categories associated with posts in */r/Lonely* and */r/depression*, respectively.

**Table 11:** Results from LIWC categories associated with posts in */r/Lonely* compared to posts in */r/depression* by the same users.

LIWC category	Cohen’s D
Social processes	0.535
Friends	0.384
Leisure	0.180

**Table 12:** Results from LIWC categories associated with posts in */r/depression* compared to posts in */r/Lonely* by the same users.

LIWC category	Cohen’s D
Health	0.351
Death	0.213
First person singular pronoun	0.197
Anger	0.196

### 6.3 Results

It was observed that in the loneliness forum, these users tend to publish posts on topics related to relationships and talking with friends (Table 9) and they tend to use words associated with the LIWC categories on friendship and leisure (Table 11). However, in the depression forum, these users tend to publish posts on topics related to mental health, self-harm, and issues with sleep (Table 10) and they tend to use words associated with the LIWC categories on health, death, first person singular pronoun, and anger (Table 12).

## 7 Compare posts in */r/Lonely* by */r/Lonely* users only to */r/Lonely* + */r/depression* users

In this section, similar to sections **4** and **5** using LDA and LIWC, the differences in language use in posts published in */r/Lonely* by users who are active members of both */r/Lonely* and */r/depression* compared to users who are active members of */r/Lonely* but not active members of */r/depression* is determined. The dataset used for the analysis in this section is described in Table 4.

### 7.1 LDA

Similar to sections **4.1** and **5.1**, using LDA [8], 20 topics are generated using posts from */r/Lonely* by users who are either active members of */r/Lonely* and */r/depression* or are active members of */r/Lonely* but not members of */r/depression* and topics which frequently occur in posts published by users who belong to these groups are identified, as shown in Tables 13 and 14.

**Table 13:** Topics in */r/Lonely* associated with Lonely users only.

LDA Topics and highly correlated words	Cohen’s D
work, job, home, years, moved, live, working, months, ago, city	0.127
talk, lonely, chat, message, post, hey, pm, wanna, you’re, reddit	0.104

**Table 14:** Topics in */r/Lonely* associated with Lonely and depressed users.

LDA Topics and highly correlated words	Cohen’s D
i’m, don’t, i’ve, it’s, can’t, i’ll, that’s, anymore, there’s, doesn’t	0.113
love, loved, miss, happy, night, dream, hug, hold, heart, give	0.10

### 7.2 LIWC

Here, LIWC is used to determine the differences in the use of LIWC categories between posts published in */r/Lonely* by users who are either active members of both */r/Lonely* and */r/depression* or are active members of */r/Lonely* but not members of */r/depression*. Table 15 shows the LIWC categories associated with */r/Lonely* posts by users who post in both */r/Lonely* and */r/depression*. *“Family”* (Cohen’s D = 0.10) was the only LIWC category most associated with */r/Lonely* posts by users who post in */r/Lonely* but not in */r/depression*.

**Table 15:** Results from LIWC categories associated with */r/Lonely* posts by users who post in both */r/Lonely* and */r/depression* compared to posts in */r/Lonely* by users who post in */r/Lonely* but not in */r/depression*.

LIWC category	Cohen’s D
Negation	0.136
Focus present	0.116
Anger	0.106
Death	0.104

### 7.3 Result

We observed that on the loneliness forum */r/Lonely*, users who are members of the loneliness forum but not members of the depression forum tend to post about topic themes related to work and wanting to chat with others (Table 13) and tend to use words associated with the LIWC category on *“Family”*. Users who are members of both the loneliness and depression forums, tend to publish loneliness forum posts on topic themes related to affection relative to relationships (Table 14) and use more words associated with the LIWC categories on negation, focus present, anger, and death (Table 15).

## 8 Discussion

In this section, the findings from this work are summarized.

Regarding the analysis between */r/Lonely* and */r/AskReddit* forum posts, it was observed that in the loneliness forum, users tend to make reference to issues with socializing in school/college; this is consistent with prior work which determined that loneliness may affect the ability of students to socialize in college [38]. Also, posts in the loneliness forum tend to make more reference to self i.e. “first person singular pronouns” this finding aligns with prior work [3], which demonstrated that users who self-express loneliness on social media tend to use more personal-pronouns in their social media posts when compared to a control group. Other findings in the analysis in this section show that in loneliness forums users tend to use more negation words and words associated with sadness. Also, loneliness forum posts tend to make reference to topic themes about time (e.g. time, day, today, ago) relative to relationships, which may indicate that in the loneliness forum, users tend to post about time spent with family and friends.

In the advice seeking forum, */r/AskReddit*, users tend to ask more questions, post about songs and movies, and use words associated with socializing and leisure. Also, in the advice seeking forum, these users tend to seek advice/ask questions on topic themes related to relationships.

The findings around the analysis between the posts on */r/Lonely* and */r/AskReddit* may aid in the design of online interventions around loneliness. For example, given that in the advice seeking forum, users tend to seek advice on topics related to relationships, an online intervention may aim to help teach users who express loneliness on online forums some social skills or ways in which they could be successful in seeking, finding, and maintaining relationships.

Posts published by the same users in the loneliness and depression forums, respectively were compared and it was observed that in the loneliness forum, these users tend to publish posts on topic themes related to relationships and talking with friends. However, in the depression forum, these users publish posts on topic themes related to self-harm, issues with sleep, and mental health.

The finding around issues with sleep is in line with prior work, which linked poor sleep quality with depression [39]. Also, in [3], it was determined that individuals who express loneliness on social media discuss sleep deprivation.

The differences in the topic themes in loneliness forum posts by users who are members of both the loneliness forum and the depression forum compared to users who are members of the loneliness forum but not members of the depression forum were identified. It was observed that users who are members of the loneliness forum but not members of the depression forum tend to, for example, post about topic themes related to work/job; this may suggest that some of these users spend a lot of their time at their jobs and do not have time to socialize or make friends. Also, these users post about wanting to chat with members of the forum and use words associated with the LIWC categories on socializing, friendships, and leisure.

Users who are members of both the loneliness and depression forums tend to use more words associated with the LIWC categories on negation, anger, focus on the present, and death and publish posts on topic themes associated with affection relative to relationships.

These findings around the analysis on loneliness forum posts by users who are members of both the loneliness and depression forums compared to posts by users who are members of the loneliness forum but not members of the depression forum show that online interventions related to loneliness should be cognizant with the differences in language use in loneliness forum posts by users who belong to these groups. For example, interventions designed for users who are lonely but not depressed may provide tips on how to make friends and links to events that might be of interest to them such as concerts or meetings of a local gardening club (i.e. if it is known where the user resides). The interventions for users who may be lonely and depressed may in addition to the interventions for users who are lonely but not depressed, provide professional mental health services.

## 9 Limitations

The data used for these analyses are from Reddit users and is not representative of the general population. Also, it is possible that some active members of */r/Lonely* who may be depressed do not publish posts in */r/depression*, hence this work cannot infer about these users.

## 10 Conclusion and future work

In this work, the language use differences in loneliness forum posts and posts (published around the same time period) in other forums by the same users are determined. The findings from this work show that these language use differences reflect the differences in support needs/concerns expressed in posts in these forums by these users. These findings can help guide and inform the design of online interventions around loneliness. Prior work [40] determined that in online forums focused on health and well-being, users seek and give social supports such as emotional and informational supports; a future research work to explore is to study the types of social supports which are sought and given in loneliness forum posts compared to posts (published at the same time period) by the same users in other forums.

## Supporting information

S1 File */r/Lonely* forum posts associated with users who are members of */r/Lonely* but not members of */r/depression* and those who are members of both */r/Lonely* and */r/depression*. (CSV)

S2 File */r/Lonely* forum posts and */r/depression* forum posts by the same users. (CSV)

S3 File */r/Lonely* forum posts and */r/AskReddit* posts by the same users. (CSV)
